# A Pragmatic Comparison Between Aerobic Exercise and Suryanamaskar in Stress Management in Medical Professionals: A Quasi-experimental Study

**DOI:** 10.7759/cureus.29414

**Published:** 2022-09-21

**Authors:** Sharvari J Joshi, Sabih N Khan, Jeet S Kantharia, Shrikant Mhase, Aishwarya A Pashine, Roshan Umate

**Affiliations:** 1 Rehabilitation, Mahatma Gandhi Mission (MGM) School of Physiotherapy, Aurangabad, IND; 2 Cardiorespiratory Physiotherapy, Mahatma Gandhi Mission (MGM) School of Physiotherapy, Aurangabad, IND; 3 Community Physiotherapy, Mahatma Gandhi Mission (MGM) School of Physiotherapy, Aurangabad, IND; 4 Research, NKP Salve Institute of Medical Sciences and Research Centre, Nagpur, IND; 5 Research and Development, Datta Meghe Institute of Medical Sciences, Wardha, IND

**Keywords:** quasi-experimental study, fitt, yoga, medical professionals, suryanamaskar, aerobic exercise, stress

## Abstract

Background

Stress is an episodic process in an individual’s life that depends upon the circumstances that elicit this process, later jeopardizing an individual’s mental balance and leading to depression and anxiety. Yoga is a traditional component of physical activity that contains three main domains, namely, maintaining the correct posture (asanas), control over one’s breath (pranayama) and meditation with complete concentration (dhyana), which are proven to be effective in enhancing the psychological, physical, and spiritual wellbeing of an individual along with mindfulness. The purpose of the present study was to compare aerobic exercise (AE) and Suryanamaskar (SN) in terms of the best intervention in lowering the level of stress in medical professionals (MPs).

Methods

A quasi-experimental study including a pragmatic comparison was conducted involving 30 participants who were divided into two groups A and B. The participants in group A did treadmill walking and the participants in group B were made to perform the complete cycle of SN with all the 12 asanas for four weeks.

Results

The outcomes demonstrated that both AE and SN had significant statistical values in terms of pre- and post-intervention Perceived Stress Scale (PSS) scores, resting heart rate, and systolic and diastolic blood pressure. Also, when post-intervention mean values of both the groups were compared for all the parameters, only mean PSS values were found to be statistically significant.

Conclusion

The current study concluded that both AE and SN were beneficial in decreasing the level of stress in MPs with SN being statistically more significant in reducing stress levels than AE. Both interventions are safe to perform.

## Introduction

Stress is a broad term causing a pessimistic impact on the physical, mental, and emotional state of an individual, it is a psycho-physiological process that acts as an antagonist in an individual’s emotional behaviour [1]. Stress is an episodic process in an individual’s life that depends upon the circumstances that elicit this process, later jeopardizing an individual’s mental balance and causing disorders such as depression and anxiety [2]. Medical professionals (MPs) include doctors, surgeons, and nurses who deal with a huge amount of emotional and mental stress in their day-to-day lives with respect to their occupation, which can lead to secondary insults to their psychological behaviour [3].

Physical activity (PA) is an umbrella term that involves various activities that help in developing physical, cardiovascular as well as psychological functions in a human body causing a progressive momentum in the skeletal muscles of the body [4]. Aerobic exercise (AE) also called endurance training is a renowned part of PA, inclusive of treadmill walking, running, jogging, swimming, etc., and is mentioned in terms of frequency, intensity, time, and type of exercise, known as the FITT principle [5].

Yoga, introduced by the ancient Indian culture, is a traditional procedure performed in order to promote strength, flexibility, and endurance and helps in increasing physical and mental health. Yoga is a traditional component of PA that contains three main domains, namely, maintaining the correct posture (asanas), control over one’s breath (pranayama), and meditation with complete concentration (dhyana), which are proven to be effective in improving the physical, mental, and spiritual health of an individual along with mindfulness [6]. Suryanamaskar (SN) is an ancient yogic exercise sequence of worshiping the Sun (Surya meaning Sun, and namaskar, meaning salutation in honor) in different postures or asanas in rhythmic breathing emphasizing on the overall stretch and force on the body that promotes physical fitness and mental health [7]. SN is a cycle of 12 steps, each carried along with inspiration and expiration wherever required, helping in improving the respiratory capacity and also enhancing the haemodynamic vitals of an individual. Other benefits involve improvement in digestion, promoting good excretory function, maintaining mental peace, and helping in relaxation [8].

The various asanas of SN are named according to their scientific significance with each asana providing a remarkable benefit to the body [9]. The positive effect of AE on stress has been proven in various studies previously, and also, the impact of SN over stress has been found to be positive [10,11]. Therefore, this study was carried out to find out the more effective method between AE and SN in relieving stress in MPs.

## Materials and methods

A quasi-experimental study with random sampling was conducted at the cardio-respiratory department in Pravara Institute of Medical Sciences (PIMS) after receiving permission from the Institutional Ethics Council (IEC no. PIMS/CPT/IEC/2017/183); the four-week study included 30 subjects. This study was inclusive of the subjects who were willing to participate, including both the genders, who were an MP by occupation and who scored 14 to 26 on the Perceived Stress Scale (PSS). Subjects with any underlying medical or psychiatric illness along with any disorder of the musculoskeletal and neurological system were excluded from this study. After the selection, signed informed consent was collected from every participant.

The participants were divided into two groups A and B consisting of 15 participants each. The allocation of the participants in the groups was done on the basis of the coin toss method; the materials and equipment used were a treadmill, sphygmomanometer, Polar heart rate monitor (Polar Electro, Finland), pulse oximeter, yoga mat, and pre- and post-data collection sheet.

In the pre-data collection sheet, demographic data and the general physical examination including blood pressure, PSS documentation, and resting heart rate (RHR) values for both the groups were recorded [12-15]. Following this, the participants in both the groups were introduced to their respective interventions. Group A performed AE on the basis of the FITT principle wherein the frequency was five days per week, the intensity was 40%-60% of HRmax (HRmax = 220 - Age), for 30 minutes, and the type of exercise was endurance or aerobic training on a treadmill prior to which a six-minute walk test, a submaximal exercise testing, was done to check the cardiopulmonary endurance; post-intervention, the rate of perceived exertion was calculated by the modified Borg scale. Group B performed all the 12 asanas of SN every five days for four weeks and the duration was 30 minutes (Table 1). Pre-intervention, the subjects were made to rest for five minutes and then the RHR was measured whereas, post-intervention, the subjects were advised to rest for 120 seconds, and later the RHR was assessed.

**Table 1:** Twelve asanas of Suryanamaskar and their benefits Ref. [9]

Number of pose	Asanas	Benefits
I	Pranamasana	Helps in maintaining the balance, relaxes the body and concentrates on the breathing
II	Hasta Uttanasana	Improves the digestion and elicits the flexibility of back and hip along with arms and shoulder
III	Hastapaadasana	Improves the hemodynamic vitals along with lymphatic system and also increases the flexibility of the back and leg with facilitation of spinal nerves
IV	Ashwa Sanchalanasana	Strengthens the hand, wrist and back muscles
V	Adho Mukha Parvatasana	Promotes circulation of blood along with strengthening of hand and wrist musculature and helps in stress relief of the neck
VI	Ashtanga Namaskara	Strengthens all the musculature of the body and increases the flexibility of the body
VII	Bhujangasana	Promotes circulation to abdominal organs and improves the function of respiration and digestion
VIII	Adho Mukha Parvatasana	Promotes circulation of blood along with strengthening of hand and wrist musculature and helps in stress relief of the neck
IX	Ashwa Sanchalanasana	Strengthens the hand, wrist and back muscles
X	Hastapaadasana	Improves the hemodynamic vitals along with lymphatic system and also increases the flexibility of the back and leg with facilitation of spinal nerves
XI	Hasta Uttanasana	Improves the digestion and elicits the flexibility of back and hip along with arms and shoulder
XII	Pranamasana	Helps in maintaining the balance, relaxes the body and concentrates on the breathing

**Figure 1:**
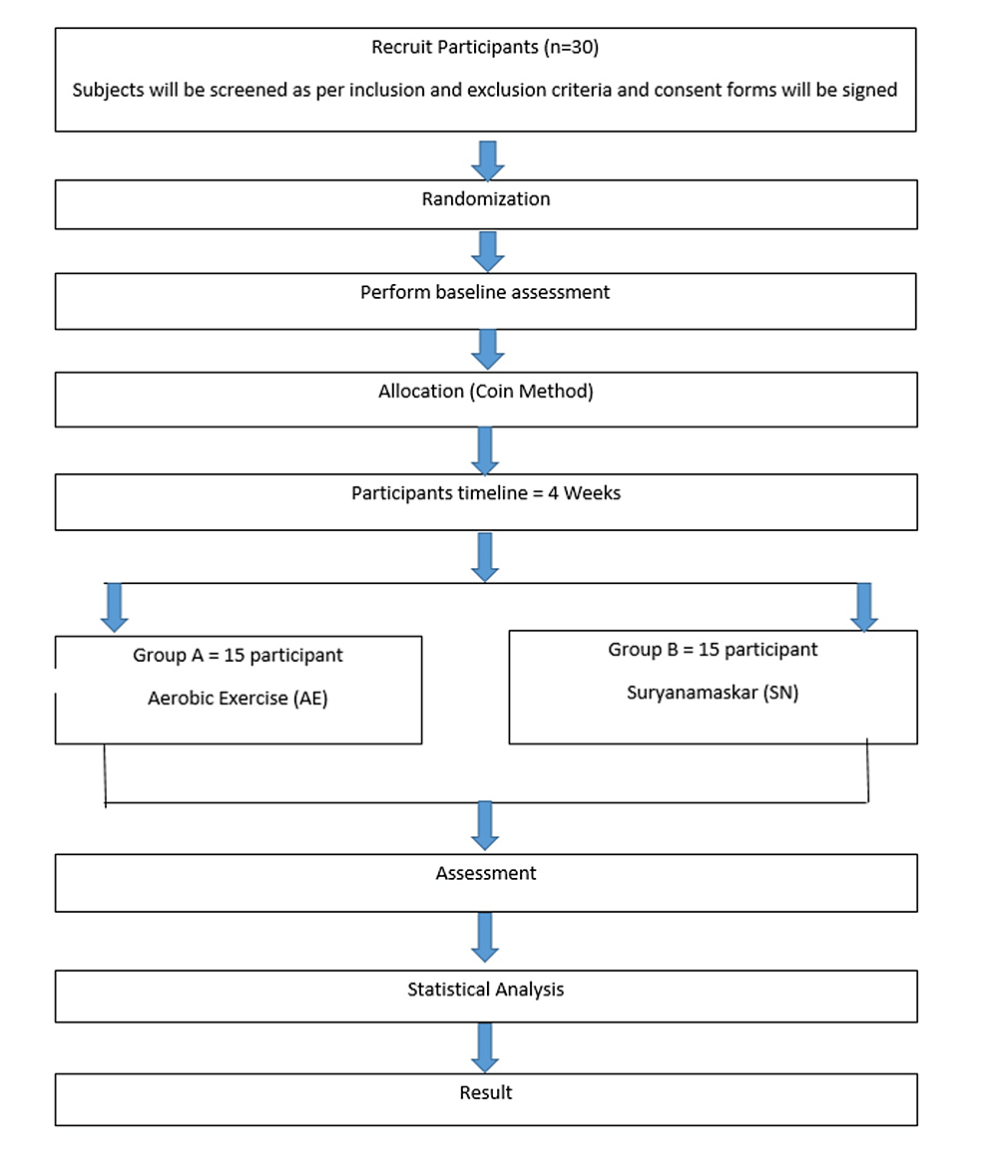
Flow chart of the procedure

Also, the Polar heart rate monitor device, which has sensor technology, was used for the estimation of the heart rate and was worn on the chest throughout the SN cycle and AE by the participant. After the completion of the interventions, again all the parameters were assessed and documented in the post-data collection sheet. The flowchart of the general procedure is described in Figure 1.

## Results

The current study was conducted chiefly to compare AE and SN, to find the more effective intervention in stress among the two. After the termination of the four-week protocol, when the pre- and post-intervention PSS mean values were compared for AE and SN individually, the results were statistically extremely significant, which demonstrated that both AE and SN proved beneficial in reducing the level of stress in MPs, with SN statistically being more beneficial (Table 2).

**Table 2:** Pre- and post-intervention PSS mean values for AE and SN AE = aerobic exercise, SN = Suryanamaskar, SD = standard deviation, PSS = Perceived Stress Scale

Intervention	Pre-intervention PSS, mean±SD	Post-intervention PSS, mean±SD	p-value	t-value	Result
AE	27.33±2.637	19.066±2.219	0.0001	22.271	Extremely significant
SN	26.933±4.044	16.2±3.858	0.0001	24.928	Extremely significant

When the mean values for pre-intervention RHR and post-intervention RHR were compared for AE and SN individually, the results were statistically extremely significant in groups A and B, as described in Table 3.

**Table 3:** Pre- and post-intervention mean values for resting heart rate for AE and SN AE = aerobic exercise, SN = Suryanamaskar, SD = standard deviation

Intervention	Pre-intervention, mean±SD	Post-intervention, mean±SD	p-value	t-value	Result
AE	86.13±5.317	81.8±6.002	0.0001	16.037	Extremely significant
SN	87.4±5.591	82.26±5.499	0.0001	14.992	Extremely significant

Both AE and SN were found to be beneficial in reducing diastolic blood pressure (DBP) and systolic blood pressure (SBP) in groups A and B (Tables 4, 5).

**Table 4:** Pre- and post-intervention mean values for diastolic blood pressure for AE and SN AE = aerobic exercise, SN = Suryanamaskar, SD = standard deviation

Intervention	Pre-intervention, mean±SD	Post-intervention, mean±SD	p-value	t-value	Result
AE	81.2±3.764	79.3±3.117	0.0013	3.674	Very significant
SN	81.2±3.764	78.93±1.981	0.0008	3.900	Very significant

**Table 5:** Pre- and post-intervention mean values for systolic blood pressure for AE and SN AE = aerobic exercise, SN = Suryanamaskar, SD = standard deviation

Intervention	Pre-intervention, mean±SD	Post-intervention, mean±SD	p-value	t-value	Result
AE	123.6±5.616	119.33±5.851	0.0001	13.514	Extremely significant
SN	122.66±5.88	119.2±5.457	0.0001	8.404	Extremely significant

When post-AE mean values were compared with post-SN values, for PSS, RHR, SBP, and DBP, statistical significance was found only for PSS, with SN being more significant statistically (Table 6).

**Table 6:** Comparison between post-AE and post-SN mean values for PSS AE = aerobic exercise, SN = Suryanamaskar, PSS = Perceived Stress Scale, SD = standard deviation

AE versus SN (PSS)					
	Post-intervention (AE)	Post-intervention (SN)	p-value	t-value	Result
Mean±SD	19.06±2.219	16.2±3.858	0.0188	2.495	Significant

## Discussion

The current study was carried out to find out the effectiveness of AE in comparison to SN in managing stress in MPs following which the outcomes of this pragmatic quasi-experimental study proved extremely significant for both the interventions. When the mean values of pre- and post-intervention PSS were compared for AE and SN individually, the results were statistically extremely significant demonstrating that both AE and SN proved beneficial in reducing the level of stress in MPs, with SN statistically being more beneficial. A previous study demonstrated that exercise helped in alleviating anxiety, reducing stress levels, lessening symptoms of depression, and enhancing mental well-being [16]. Catecholamines secreted from the central adrenal gland have a close relationship with the functions of the sympathetic nervous system physiologically. Increased levels of catecholamines are apparently important facilitators of exercise functions [17]. A recent study described that past research documents SN as an effective intervention in improving psychological variables like a sense of well-being and feeling of relaxation; it concluded that SN plays a positive and significant role in decreasing stress levels as it improves relaxation by reducing sympathetic activity and improves the sense of general well-being by increasing parasympathetic activity [1].

The second component considered was resting heart rate; when the mean values for pre-intervention and post-intervention RHR were compared for AE and SN individually, the results were statistically extremely significant, showing that both AE and SN proved beneficial in reducing RHR values in MPs, which was in congruence with a study that showed AE as an effective intervention in reducing RHR and increasing physical fitness [18]. Additionally, the results of a previous study demonstrated a significant decrease in the RHR in all the three groups performing SN, pranayama, and a combination of both; SN enhances the cardio-vascular functions partially, because of its favorable effects on aerobic capacity and increases cardio-vascular loading, which in turn improves stroke volume. The lower the resting heart rate, the better the ability of the heart to withstand stressful stimuli and still stay calm [19].

The third parameter taken into account was blood pressure including systolic and diastolic blood pressure; pre- and post-intervention mean values for AE and SN individually were found to be significant for both, showing that both AE and SN are beneficial in reducing SBP and DBP. In a study by Wen and Wang, a significant reduction in SBP and DBP was identified in the aerobic group after AE training when compared with the control group. One explanation for this reduction effect was that regular AE prevents the age-associated vascular endothelial dysfunction; an experiment in rats showed that aerobic exercise training can reduce blood pressure by improving vascular stiffness and endothelial function [20]. Similarly, a meta-analysis concluded that yoga was associated with a modest but significant reduction in blood pressure (≈4 mmHg, systolic and diastolic) and can be preliminarily recommended as an effective intervention for reducing blood pressure as it increases cardiovascular loading, which in turn improves stroke volume [21].

However, when post-AE mean values were compared with post-SN values, including PSS, RHR, SBP, and DBP, statistical significance was found only for PSS, showing that both AE and SN had a positive impact on reducing stress levels in MPs with SN being more significant statistically; in the case of other parameters, no statistical significance was found when both post-AE and post-SN mean values were compared. AE and SN showed the same level of improvement in reducing stress levels, and cardiorespiratory endurance. Hence, according to an individual’s interest, he/she can go for either of the protocol. But as the participants were MPs and stress levels in their day-to-day life are extremely significant, a comparison of the mean values showed that SN proved more beneficial than AE. Along with the parameters considered in this study, AE and SN also proved beneficial in maintaining the range of inflammatory mediators, improving mental disorders, reducing the frequency of migraine attacks, decreasing adipose tissue, and in treating premenstrual syndrome [22-27].

The future scope of this study can involve a larger sample size as this study involved only 30 participants; also, the duration of the intervention, which was four weeks in this study, can be increased. Additionally, this study only included MPs; further studies can include various other professionals, from different age groups with various other parameters, and other forms of AE and yogic procedures.

## Conclusions

We found that both the interventions AE and SN proved extremely significant in reducing stress levels, RHR, SBP, and DBP in accordance to their own benefits with SN statistically being more beneficial in reducing stress than AE. However, the choice of intervention ultimately depends on the medical professional according to one's convenience of performing the intervention, period of intervention, and analysis of intervention. Hence, both interventions are safe to perform.

## References

[1] Agre S, Agrawal R, Ishrat S (2021). Effect of Suryanamaskar on stress levels in SSC students. Indian J Public Health Res Dev.

[2] Gala D, Savalia JK (2022). Effect of Suryanamaskar on stress in delayed-postpartum Indian women: a pilot study. Dev Sanskriti Interdiscip Int J.

[3] Kriakous SA, Elliott KA, Lamers C, Owen R (2021). The effectiveness of mindfulness-based stress reduction on the psychological functioning of healthcare professionals: a systematic review. Mindfulness.

[4] Schultchen D, Reichenberger J, Mittl T, Weh TR, Smyth JM, Blechert J, Pollatos O (2019). Bidirectional relationship of stress and affect with physical activity and healthy eating. Br J Health Psychol.

[5] Herbert C, Meixner F, Wiebking C, Gilg V (2020). Regular physical activity, short-term exercise, mental health, and well-being among university students: the results of an online and a laboratory study. Front Psychol.

[6] Wang W, Long F, Wu X, Li S, Lin J (2022). Clinical efficacy of mechanical traction as physical therapy for lumbar disc herniation: a meta-analysis. Comput Math Methods Med.

[7] Kodidala SR, Raj Hans PS, Sorout J, Soni H (2022). Impact of Suryanamaskar training on cardiovascular, respiratory, and cognitive functions among medical students. Avicenna J Neuro Psycho Physiology.

[8] Hipparagi M, Gangadhar P (2019). Suryanamaskar for human wellness. Int J Phys Educ Sports Health.

[9] Sachan A, Solanki G (2021). Surya namaskar: its techniques and health benefits. Indian J Nat Sci.

[10] Hives BA, Buckler EJ, Weiss J, Schilf S, Johansen KL, Epel ES, Puterman E (2021). The effects of aerobic exercise on psychological functioning in family caregivers: secondary analyses of a randomized controlled trial. Ann Behav Med.

[11] Adhikari DA (2021). Effect of yogic exercises on stress and aggression among the adolescents. Int J Yogic Hum Mov Sports Sciences.

[12] Walker KA, Sharrett AR, Wu A (2019). Association of midlife to late-life blood pressure patterns with incident dementia. JAMA.

[13] Huang F, Wang H, Wang Z (2020). Psychometric properties of the perceived stress scale in a community sample of Chinese. BMC Psychiatry.

[14] Manzar MD, Salahuddin M, Peter S, Alghadir A, Anwer S, Bahammam AS, Pandi-Perumal SR (2019). Psychometric properties of the perceived stress scale in Ethiopian university students. BMC Public Health.

[15] Quer G, Gouda P, Galarnyk M, Topol EJ, Steinhubl SR (2020). Inter- and intraindividual variability in daily resting heart rate and its associations with age, sex, sleep, BMI, and time of year: Retrospective, longitudinal cohort study of 92,457 adults. PLoS One.

[16] Mikkelsen K, Stojanovska L, Polenakovic M, Bosevski M, Apostolopoulos V (2017). Exercise and mental health. Maturitas.

[17] Mohebbi Z, Dehkordi SF, Sharif F, Banitalebi E (2019). The effect of aerobic exercise on occupational stress of female nurses: a controlled clinical trial. Invest Educ Enferm.

[18] Kang SJ, Kim EH, Ko KJ (2016). Effects of aerobic exercise on the resting heart rate, physical fitness, and arterial stiffness of female patients with metabolic syndrome. J Phys Ther Sci.

[19] Singh A, Sarika Sarika, Sandhu JS (2011). Combined effects of Yogkriyas and Pranayama on heart rate variability and resting heart rate. J Res Educ Indian Med.

[20] Wen H, Wang L (2017). Reducing effect of aerobic exercise on blood pressure of essential hypertensive patients: a meta-analysis. Medicine (Baltimore).

[21] Hagins M, States R, Selfe T, Innes K (2013). Effectiveness of yoga for hypertension: systematic review and meta-analysis. Evid Based Complement Alternat Med.

[22] Zheng G, Qiu P, Xia R, Lin H, Ye B, Tao J, Chen L (2019). Effect of aerobic exercise on inflammatory markers in healthy middle-aged and older adults: a systematic review and meta-analysis of randomized controlled trials. Front Aging Neurosci.

[23] Taheri M, Irandoust K, Mirmoezzi M, Ramshini M (2018). Effect of aerobic exercise and omega-3 supplementation on psychological aspects and sleep quality in prediabetes elderly women. Sleep Hypn.

[24] Lemmens J, De Pauw J, Van Soom T (2019). The effect of aerobic exercise on the number of migraine days, duration and pain intensity in migraine: a systematic literature review and meta-analysis. J Headache Pain.

[25] Prasanna Venkatesh L, Vandhana S (2022). Insights on Surya namaskar from its origin to application towards health. J Ayurveda Integr Med.

[26] Srivastav R, Hyanki D, Chaurasia P, Bhardwaj A (2020). Effect of Surya Namaskar on high sensitive C-reactive protein levels in overweight and obese middle aged adults. Int J Clin Exp Physiol.

[27] Vaghela N, Mishra D, Sheth M, Dani VB (2019). To compare the effects of aerobic exercise and yoga on premenstrual syndrome. J Educ Health Promot.

